# Prevalence of depressive symptoms among Hispanic/Latino ethnic subgroups during the COVID-19 pandemic

**DOI:** 10.1038/s41598-024-57064-4

**Published:** 2024-03-20

**Authors:** Maryam Elhabashy, Jolyna Chiangong, Kevin Villalobos, Francisco A. Montiel Ishino, David Adzrago, Faustine Williams

**Affiliations:** 1 Division of Intramural Research, National Institute on Minority Health and Health Disparities, 11545 Rockville Pike, Bethesda, MD USA; 2 Division of Intramural Research, National Institute of Environmental Health Sciences, National Institutes of Health, Durham, NC USA

**Keywords:** Risk factors, Public health, Epidemiology

## Abstract

Hispanic/Latino populations experienced disproportionate exposure to depression risk factors during the COVID-19 pandemic. While aggregated data confirm the risks of depressive symptoms among Hispanic/Latino individuals, little research uses disaggregated data to investigate these risks based on ethnic subgroups. Using the “Understanding the Impact of the Novel Coronavirus (COVID-19) and Social Distancing on Physical and Psychosocial (Mental) Health and Chronic Diseases” survey, which was distributed nationally between May 13, 2021, and January 9, 2022 (N = 5413), we estimated the prevalence of depressive symptoms among Hispanic/Latino ethnic subgroups during the pandemic. We performed descriptive analysis on a 116-item survey, which collected disaggregated data from Hispanic/Latino individuals aged ≥ 18 years (n = 1181). About one-third of the participants reported depressive symptoms (31.3%), with those who self-identified as other Hispanic/Latino/Spanish origin (40.2%) reporting the highest depressive symptom prevalence. Among participants who reported depression treatment before the pandemic, the highest reports of treatment were among Puerto Rican (81.8%) participants. More than one-third of participants receiving prior depression treatment (38.7%) reported treatment interference by the pandemic, mostly among Central American individuals (50.0%). This study highlights the need for integrating more disaggregated data into public health approaches which seek to target population subgroups and reduce racial/ethnic mental health disparities.

## Introduction

The COVID-19 pandemic has increased (and continues to increase) stress in many populations, affecting their mental health. Evidence suggests that pandemics increase the risk of developing mental disorders and poor mental well-being^1^. The percentage of United States (US) adults with symptoms of anxiety or depressive disorder increased from 36.4% to 41.5% between August 2020 and February 2021^2^. In particular, compared to pre-pandemic, the prevalence of depressive symptoms and major depressive disorder has risen since the COVID-19 pandemic began^3–5^. Depression is one of the most common mental health disorders^6^. It is characterized by persistently low or depressed mood, decreased interest in pleasurable activities, feelings of guilt or worthlessness, poor concentration, sleep disturbances, or thoughts of suicide^7^. Therefore, the rise in depression during the COVID-19 pandemic is a major concern that needs to be further evaluated by identifying the population subgroups most likely to be affected in a similar situation.

The prevalence of depressive symptoms in the US was more than threefold higher during the COVID-19 pandemic compared to before the pandemic^4^. Moreover, research shows that communities of color have been disproportionately impacted by the COVID-19 pandemic^8–10^. The National Institute of Mental Health has reported that major life changes, trauma, and stress are significant risk factors for depression^11^. Given the overall increased rates of depression and its risk factors since the advent of COVID-19, it is crucial to examine and understand unique experiences among ethnic minority communities, including Hispanic/Latino populations, where risk factors may be more prevalent and their implications more severe. During the COVID-19 pandemic, Hispanic/Latino populations experienced disproportionate exposure to stressors and COVID-19 risk factors^12^. These factors include being essential workers, residing in dense neighborhoods and larger households, and facing difficulties in maintaining optimal social distancing, which increase the risk of psychological distress and depression^13–15^.

The effects of the COVID-19 pandemic have exacerbated several determinants of mental health^3^, including discrimination, socioeconomic status (SES), familial relationships, poor housing quality and housing instability, employment, and job insecurity^16–18^. Nearly eight million (18.4%) Hispanic/Latino adults reported having a mental illness during the early stages of the pandemic in 2020^19^. Moreover, it has been observed that Hispanic/Latino individuals are experiencing greater COVID-19-related stress when compared to non-Hispanic/Latino White persons^9,20^. While studies during the pandemic have shown a clear risk of depressive symptoms among Hispanic/Latino individuals, few have examined this from a Hispanic/Latino ethnic subgroup perspective, which could more clearly identify group differences^21–23^. This current descriptive analysis addresses this research gap by estimating the prevalence of depressive symptoms among US Hispanic/Latino ethnic subgroups during the COVID-19 pandemic using a nationally distributed survey. Current studies exploring Hispanic/Latino populations are limited, and become more so by subgroup range and availability^24^. As such, our aim for this descriptive study, which is exploratory in nature, is to aid in hypothesis generation. Also, by disaggregating population data, this study provides a more comprehensive understanding of Hispanic/Latino population health and enables the design of more tailored and specific interventions/recommendations.

## Methods

The “Understanding the Impact of the Novel Coronavirus (COVID-19) and Social Distancing on Physical and Psychosocial (Mental) Health and Chronic Diseases” survey was distributed nationally between May 13, 2021, and January 9, 2022, and was cross-sectional in design. Qualtrics LLC was contracted to facilitate recruitment and distribution of the web-based survey, distributing 10,000 surveys to US-born (i.e., Hispanic/Latino, White, Black, Asian, American Indian/Alaskan Native, Native Hawaiian/Pacific Islander) and foreign-born (i.e., African, Middle Eastern, Hispanic/Latino, Asian) individuals. Oversampling was applied for low-income and rural adults. The research support firm, Information Management Services, Inc., cleaned and managed the deidentified survey data. Further information regarding the survey design and implementation has been reported elsewhere^25^. The 116-item survey collected data from multiple domains, including but not limited to the impacts of the COVID-19 pandemic, mental health, acculturation, demographic, and socioeconomic factors. Of the 5938 surveys returned, 5413 (91.16%) were deemed complete and usable for analysis after expert review and fraud detection (Qualtrics and Information Management Services, Inc.). The Hispanic/Latino subsample of 1181 participants was used for this descriptive study.

Descriptives were tabulated from the total Hispanic/Latino survey subsample, and then by Hispanic/Latino subgroups (i.e., Mexican/Mexican American, Puerto Rican, Cuban/Cuban American, Dominican, Central American, South American, other Hispanic/Latino/Spanish origin group). Chi-squared tests with *p*-values were used to determine statistically significant differences in the prevalence of depressive symptoms by sociodemographic characteristics (Table 1). Mental health questions included self-reported general mental health, effects of COVID-19 pandemic on depression treatment (based on self-reported responses of whether there was treatment prior to and during the pandemic), and Patient Health Questionaire-2 (PHQ-2). PHQ-2 was categorized as either no depressive symptoms or depressive symptoms based on scores of 0 to 2 or 3 to 6, respectively, for the following two items: whether over the last 2 weeks they experienced (1) “Little interest or pleasure in doing things”; and (2) Feeling down, depressed, or hopeless”. The following responses were scored accordingly: (1) “not at all” for a score of 0; (2) “several days” for a score of 1; (3) “more than half the days” for a score of 2; and (4) “nearly every day” for a score of 3.

**Table 1:** Hispanic/Latino descriptive statistics during the COVID-19 pandemic.

	Hispanic/Latino ethnic subgroups																	
	Mexican/Mexican American		Puerto Rican		Cuban/Cuban American		Dominican		Central American		South American		Other Hispanic/Latino/Spanish origin		Total		x^2^ (df)	*p*-value
	n	%	n	%	n	%	n	%	n	%	n	%	n	%	N	%		
	533	45.1	189	16.0	83	7.0	67	5.7	61	5.2	143	12.1	105	8.9	1181	-		
General mental health (n = 1171)																	24.7 (18)	0.134
Poor	44	8.3	14	7.5	2	2.4	3	4.5	3	4.9	7	5.0	7	6.7	80	6.8		
Fair	102	19.3	40	21.4	17	20.7	12	17.9	13	21.3	17	12.1	24	23.1	225	19.2		
Good	183	34.6	63	33.7	23	28.1	30	44.8	15	24.6	46	32.6	31	29.8	391	33.4		
Very good/Excellent	200	37.8	70	37.4	40	48.8	22	32.8	30	49.2	71	50.4	42	40.4	475	40.6		
PHQ-2 (n = 1174)																	9.9 (6)	0.129
No Depressive Symptoms	364	68.4	123	65.4	59	71.1	46	70.8	44	72.1	110	76.9	61	59.8	807	68.7		
Depressive Symptoms	168	31.6	65	34.6	24	28.9	19	29.2	17	27.9	33	23.1	41	40.2	367	31.3		
Depression treatment before COVID-19 (n = 199)																	14.8 (6)	0.021
No treatment or other	30	32.6	8	18.2	5	35.7	4	66.7	7	50.0	10	55.6	9	52.9	70	35.2		
Depression treatment	92	67.4	44	81.8	14	64.3	6	33.3	8	50.0	18	44.4	17	47.1	199	64.8		
Depression treatment interfered by COVID-19 (n = 199)																	3.8 (6)	0.697
Did not interfere	55	59.8	26	59.1	10	71.4	4	66.7	4	50.0	14	77.8	9	52.9	122	61.3		
Interfered	37	40.2	18	40.9	4	28.6	2	33.3	4	50.0	4	22.2	8	47.1	77	38.7		
Age Category (n = 1092)																	93.9 (12)	< 0.001
18–35	269	54.1	68	40.2	23	30.7	39	66.1	35	59.3	50	37.0	57	58.2	541	49.6		
36–55	164	33.0	59	34.9	16	21.3	16	27.1	16	27.1	64	47.4	31	31.6	366	33.5		
56–85( +)	64	12.9	42	24.9	36	48.0	4	6.8	8	13.6	21	16.6	10	10.2	185	16.9		
Gender (n = 1179)																	23.6 (12)	0.012
Man	210	39.5	57	30.2	24	29.3	21	31.3	22	36.1	54	37.8	31	29.5	419	35.5		
Woman	307	57.5	131	69.3	57	69.5	41	61.2	38	62.3	86	60.1	67	63.8	726	61.6		
Something else	16	3.0	1	0.5	1	1.2	5	7.5	1	1.6	3	2.1	7	6.7	34	2.9		
Marital status (n = 1176)																	48.5 (24)	0.002
Never been married	210	39.5	57	30.5	14	17.1	31	46.3	24	39.3	42	29.6	47	45.0	425	36.1		
Living with partner	65	12.2	28	15.0	12	14.6	10	14.9	7	11.5	15	10.6	14	13.3	151	12.8		
Married	205	38.5	71	38.0	45	54.9	19	28.4	22	36.1	71	50.0	33	31.4	466	39.6		
Divorced/Separated	41	7.7	26	13.9	9	11.0	6	9.0	8	13.1	7	4.9	9	8.6	106	9.0		
Widowed	11	2.1	5	2.7	2	2.4	1	1.5	0	0.0	7	4.9	2	1.9	28	2.4		
Education (n = 1178)																	66.9 (24)	< 0.001
Less than high school	50	9.4	16	8.5	5	6.0	12	17.9	4	6.6	6	4.2	12	11.5	105	8.9		
High school or GED	147	27.6	55	29.3	13	15.7	18	26.9	14	23.0	23	16.1	29	27.9	299	25.4		
Some college, vocational or technical	178	33.5	64	34.0	29	35.0	13	19.4	21	34.4	31	21.7	28	26.9	364	30.9		
Bachelor’s degree	108	20.3	38	20.2	22	26.5	18	26.9	14	23.0	58	40.6	22	12.2	280	23.8		
Master’s degree or above	49	9.2	15	8.0	14	16.9	6	9.0	8	13.1	25	17.5	13	12.5	130	11.0		
Employment (n = 1176)																	15.3 (12)	0.226
Unemployed or nontrad. worker	221	41.6	89	47.1	40	48.8	25	37.3	20	33.3	54	37.8	45	43.3	494	42.0		
Non-essential worker	162	30.5	51	27.0	27	33.0	22	32.8	22	36.7	50	35.0	22	21.1	356	30.3		
Essential worker	148	27.8	49	26.0	15	18.3	20	29.9	18	30.0	39	27.3	37	35.6	326	27.7		
Household income (n = 1168)																	54.7 (24)	< 0.001
Less than 25,000	130	24.6	58	31.0	10	12.4	16	23.9	15	25.4	22	15.5	39	37.9	290	24.8		
25,000 to less than 35,000	89	16.8	34	18.2	18	22.2	17	25.4	12	20.3	18	12.7	20	19.4	208	17.8		
35,000 to less than 50,000	91	17.2	33	17.7	12	14.8	9	13.4	9	15.3	21	14.8	15	14.6	190	16.3		
50,000 to less than 75,000	113	21.4	37	19.8	22	27.2	11	16.4	10	17.0	32	22.5	10	9.7	235	20.1		
75,000 or more	106	20.0	25	13.4	19	23.5	14	20.9	13	22.0	49	34.5	19	18.5	245	21.0		
Years in the US (n = 1176)																	75.4 (6)	< 0.001
Less than 10 years	77	14.5	38	20.2	18	21.7	23	34.9	19	31.2	63	44.1	38	36.5	276	23.4		
10 or more years	454	85.5	150	79.8	65	78.3	43	65.2	42	68.9	80	55.9	66	63.5	900	76.5		
English speaking level (n = 1178)																	16.4 (12)	0.007
Not at all/Poor	12	2.3	10	5.3	7	8.4	5	7.5	4	6.6	14	9.8	8	7.6	60	5.1		
Fairly well	34	6.4	15	7.9	7	8.4	6	9.0	5	8.2	18	12.6	9	8.6	94	8.0		
Well/Very well	484	91.3	164	86.8	69	83.1	56	83.6	52	85.3	111	77.6	88	83.8	1024	86.9		
Vaccination (n = 1177)																	19.6 (6)	0.003
No	179	33.7	69	36.7	27	32.9	31	46.3	19	31.2	27	18.9	35	33.3	387	32.9		
Yes	352	66.3	119	63.3	55	67.1	36	53.7	42	68.9	116	81.1	70	66.7	790	67.1		

df = degrees of freedom.

Demographic information included age, gender, and marital status. Socioeconomic status information included educational attainment and employment status. Acculturation questions included linguistic acculturation based on English speaking proficiency (i.e., none or poor; fairly well; well; very well) and length of stay in the US since first arrived. COVID-19 vaccination status was also asked.

The overall research protocol of the study was reviewed by the National Institutes of Health (NIH) Institutional Review Board (IRB) for exempt review and was approved on December 23, 2020 (IRB#000308). The NIH—Intramural Research Program IRB—Human Research Protections Program—Office of Human Subjects Research Protections determined that our protocol did not involve human subjects and was excluded from IRB review. Informed consent was obtained from all subjects who participated in the survey.

## Results

As shown in Table 1, the Hispanic/Latino sample was primarily Mexican/Mexican American (45.1%), followed by Puerto Rican (16.0%), South American (12.1%), other Hispanic/Latino/Spanish origin (8.9%), Cuban/Cuban American (7.0%), Dominican (5.7%), and Central American (5.2%) participants. The majority of the participants were women (61.6%), aged 18–35 (49.6%), married (39.6%), had some college, vocational or technical education (30.9%), were unemployed or nontraditional workers (42.0%), and had a household income of less than $25,000 (24.8%). Most of the sample reported being in the US for 10 years or more (76.5%) and speak English well/very well (86.9%). When asked about COVID-19 vaccination status, the majority reported being vaccinated (67.1%).

Overall, most of the participants (74.0%) reported good and very good/excellent general mental health (33.4 and 40.6%, respectively). South American (50.4%), Central American (49.2%), and Cuban/Cuban American (48.8%) participants reported the highest proportions of very good/excellent mental health, followed by other Hispanic/Latino/Spanish origin (40.4%), Mexican/Mexican American (37.8%), Puerto Rican (37.4%), and Dominican (32.8%) participants. About 7% of the total participants reported poor general mental health, and Mexican/Mexican Americans had the highest prevalence (8.3%) within individual subgroups. This was followed by Puerto Rican (7.5%), other Hispanic/Latino/Spanish origin (6.7%), South American (5.0%), Central American (4.9%), Dominican (4.5%), and Cuban/Cuban American (2.4%) participants.

More than 31% of the total sample reported depressive symptoms. The subgroup other Hispanic/Latino/Spanish origin had the highest percentage of depressive symptoms (40.2%) followed by Puerto Rican (34.6%), Mexican/Mexican American (31.6%), Dominican (29.2%), Cuban/Cuban American (28.9%), Central American (27.9%), and South American (23.1%) participants. Within the total sample, 35.2% reported no depression treatment before the COVID-19 pandemic. The highest reports of no depression treatment before the pandemic were among Dominican participants (66.7%). This was followed by South American (55.6%), other Hispanic/Latino/Spanish origin (52.9%), Central American (50%), Cuban/Cuban American (35.7%), Mexican/Mexican American (32.6%), and Puerto Rican (18.2%) participants. In contrast, the highest reports of depression treatment before the COVID-19 pandemic were among Puerto Rican participants (81.8%), followed by Mexican/Mexican American (67.4%), Cuban/Cuban American (64.3%), Central American (50.0%), other Hispanic/Latino/Spanish origin (47.1%), South American (44.4%), and Dominican (33.3%) participants. Further, 38.7% of participants reported that COVID-19 interfered with their depression treatment. Central American participants reported the highest interference (50%), followed by other Hispanic/Latino/Spanish origin (47.1%), Puerto Rican (40.9%), Mexican/Mexican American (40.2%), Dominican (33.3%), Cuban/Cuban American (28.6%), and South American (22.2%) participants.

The majority of participants reported living in the US (acculturation) for 10 years or more (76.5%). Acculturation of 10 years or more was most prevalent among Mexican/Mexican American (85.5%) and Puerto Rican participants (79.8%). This was followed by Cuban/Cuban American (78.3%), Central American (68.9%), Dominican (65.2%), other Hispanic/Latino/Spanish origin (63.5%), and South American participants (55.9%).

## Discussion

The COVID-19 pandemic has exacerbated mental health disorder symptoms in the US population, especially in Hispanic/Latino individuals. However, there is limited information on mental health disorder symptoms among Hispanic/Latino ethnic subgroups to identify and delineate group differences to enhance tailoring public health messages and interventions. We disaggregated data to conduct descriptive analyses and estimate the prevalence of depressive symptoms among Hispanic/Latino ethnic subgroups during the COVID-19 pandemic using a sample surveyed from across the US.

We found that about one-third of the participants reported experiencing depressive symptoms. However, only 6.8% of total participants reported poor mental health. This may be indicative of stigma, prejudice, discrimination, mental health perspectives and implicit biases against individuals who struggle with mental health^26^. Past research has found that while Hispanic/Latino individuals are at high risk of depression, unmet mental health needs are often predicated on stigma, which impacts how individuals view themselves and others^26–28^. Therefore, while a tendency towards social desirability bias may have influenced how participants reported general mental health status, responses to PHQ-2 revealed that there may be a discrepancy in individual perceptions of mental health vs. individual experiences of depressive symptoms. This has important implications for overall population health when considering the comorbidities of depression, such as diabetes, cancer, and heart disease, which are among the leading causes of death among the Hispanic/Latino population^11,29^. Additionally, within the US population, Hispanic/Latino prevalences of distress, anxiety, and depression were among the highest of any racial/ethnic minority group during the COVID-19 pandemic^12,30,31^. Subsequently, there is a need for more research using disaggregated data to unravel between- and within-population subgroup mental health disparities. By specifically exploring how the Hispanic/Latino (sub)population may be disparately exposed to and impacted by depression risk factors, effective steps can be taken to mitigate such risk factors and more equitably serve the respective subgroups.

Among all individual ethnic subgroups, other Hispanic/Latino/Spanish origin and Puerto Rican participants reported the highest prevalence of depressive symptoms. These findings are consistent with other studies which have found Puerto Ricans to have a higher prevalence of psychological distress and depression when compared with other Hispanic/Latino ethnic subgroups^32,33^. This finding highlights the need to create interventions that target increased depressive symptoms within specific Hispanic/Latino subgroups, as some subgroups might be at higher risks for psychological distress and depression with the advent of large-scale societal disruptions/stressors such as the pandemic. Furthermore, the prevalence of depressive symptoms among participants of other Hispanic/Latino/Spanish origin, who may have identified as natives of various countries and/or territories, underscores the importance of data disaggregation and oversampling when exploring health disparities among minority populations. Future studies should take these considerations into account to create a more holistic understanding of the unique experiences of different Hispanic/Latino ethnic subgroups, and how their needs can be most effectively met.

The highest reports of poor general mental health were observed among Mexican/Mexican American and Puerto Rican participants. Though very limited literature has compared mental health between Hispanic/Latino subgroups, our finding regarding Mexican/Mexican Americans contrasts with literature that found them to have lower odds of psychiatric disorders than other Latino subgroups^34^. On the other hand, South American and Cuban/Cuban Americans had the highest reports of good and very good/excellent general mental health. These results may be associated with socioeconomic status and related outcomes. The 2020 US Census found that Cubans lived in households with median net worths almost double those of Mexican/Mexican Americans and Puerto Ricans^35^. More research should analyze relationships between socioeconomic statuses and related indicators (i.e., educational attainment, employment status, etc.), as well as their impacts on mental health risk exposure and resource access across Hispanic/Latino subpopulations.

More than 1 in 3 Hispanic/Latino individuals who were receiving depression treatment before the pandemic reported treatment interference due to COVID-19. While Central American individuals reported the highest rates of depression treatment interference, this subpopulation was followed closely by Puerto Rican and Mexican/Mexican American individuals. These mental health disparities, compounded by the fact that Puerto Rican and Mexican/Mexican American persons reported the highest rates of depression treatment pre-pandemic^36–38^ as well as the highest rates of poor general mental health, are troubling. The treatment interference among Hispanic/Latino persons is concerning because mental health treatment has not decreased in the general US population. One study found that between 2015 and 2019, there were widespread increases in depression across the US, though commensurate increases in treatment were not observed^39^. However, after the onset of the pandemic in 2021, the Centers for Disease Control and Prevention (CDC) reported that the percentage of US adults receiving mental health treatment increased from 19.2 to 21.6%^40^. This implies that the observed increase in treatment occurred despite COVID-19-related interferences experienced by Hispanic/Latino individuals, highlighting the extent of disparities in mental health and access to treatment. Thus, while treatment for depressive symptoms may serve as an indicator of healthcare access, it may be a less accurate indicator of mental health burden. For instance, while Cuban participants reported the lowest rate of poor mental health, they had the third highest prevalence for depression treatment before COVID-19. Conversely, although Dominican participants reported the fourth highest rate of depressive symptoms during the pandemic, they had the lowest prevalence of depression treatment before COVID-19. These findings emphasize an increased need not only to allocate mental health resources for the Hispanic/Latino population overall but also to enhance access and outreach efforts targeting more vulnerable subpopulations.

The aforementioned findings related to Puerto Rican and Mexican/Mexican American mental health and treatment utilization (and interference) dovetail with findings related to acculturation. For all the Hispanic/Latino ethnic subgroups in our study, most participants reported living in the US for more than 10 years. The Mexican/Mexican American and Puerto Rican subgroups had the highest proportions of participants who lived in the US for more than 10 years. Reports of poor general mental health despite high levels of acculturation align with past research findings indicating that as length of stay in the US increases, immigrant health decreases^41–43^. Furthermore, while length of stay may increase assimilation and access to resources such as mental health treatment^44^, past research has found that underutilization is still common within immigrant communities. Immigrants often face barriers such as stigma, and low linguistic proficiency, and rely more on religious and social supports instead of more formal mental health treatments^38,45–47^. Previous research has also found that mental health treatment access is limited by higher rates of poverty and lower rates of insurance among Hispanic/Latino populations compared to non-Hispanic White individuals^48^. These disparities may have been exacerbated during the pandemic. Therefore, in examining potential associations between years lived in the US and prevalence of depressive symptoms among Hispanic/Latino ethnic subgroups, future research should consider cultural differences and stigma that may impact mental health treatment utilization, despite increased assimilation and access to resources over time. Accounting for such considerations in future longitudinal studies could provide additional insights into depression treatment utilization among Hispanic/Latino subgroups and support the development of more tailored mental health interventions.

Additionally, past research has found that interventions based on Western concepts of mental health conditions can lead to poorer engagement and outcomes among ethnic minorities^49–51^. Nonetheless, culturally tailored, adaptable mental health interventions are associated with greater symptom improvements among ethnic minority groups^49–51^. In the case of Hispanic/Latino populations, such adaptations have taken the form of language-matching and training therapists in values such as *respeto* (obedience to authority) and *familismo* (the concept that both nuclear and extended family are central to and more important than the individual)^50^. While such adaptations have been observed to improve outcomes, further developments in research and public health interventions should evolve to ensure that the nuanced cultural backgrounds of various ethnic subgroups can be understood and catered to in the pursuit of mental health across populations ranging from Hispanic/Latino to Asian, African, and beyond^52,53^.

There are some limitations to our study. Though our study is exploratory in nature and provides disaggregated data on Hispanic/Latino groups to aid in hypothesis-generation, the majority of the participants were Mexican/Mexican American individuals (45.1%). Thus, the results of this study are not representative or generalizable across all Hispanic/Latino subgroups in the US. Secondly, the ethnic subgroups of South American and Central American participants represent multiple countries which are not disaggregated within our results, and other ethnic subgroups had very limited survey participants. This highlights a need for oversampling of underrepresented Hispanic/Latino ethnic subgroups. Finally, the survey was only administered online and in English, potentially hindering participation based on English literacy and access to technology.

## Conclusion

Overall, this study, which relied on disaggregated data, revealed a higher prevalence of depressive symptoms among Hispanic/Latino ethnic subgroups during the COVID-19 pandemic than might have been deduced from aggregated datasets. Whereas mental health treatment received by US adults increased in general over the course of the pandemic, this study presents evidence that such treatment was largely interfered with among the Hispanic/Latino population and within more vulnerable ethnic subgroups (e.g., reporting higher prevalence of depressive symptoms and/or poor general mental health). Considering the study’s findings, further research is warranted to better understand the mental health experiences of respective ethnic subgroups within the Hispanic/Latino community. This consideration can support more effective examination and execution of culturally protective strategies, health communication, and interventions. It will be crucial to support not only the general population but also the Hispanic/Latino ethnic subgroups with the resources to mitigate mental health disorder risks and factors that may lead to depression and its comorbidities. The results of our study can be used to encourage further research on disaggregated Hispanic/Latino ethnic subgroups and increase oversampling of underrepresented populations in addressing mental health disparities in population subgroups.

## Data Availability

The data are available by making a request through Dr. FW per the new Data Management and Sharing Agreement plan.

## Abbreviations

DF: Degrees of freedom
IRB: Institutional Review Board
NIH: National Institutes of Health
PHQ-2: Patient Health Questionaire-2
SES: Socioeconomic status
US: United States
